# Automated pre-column derivatization and its application to amino-acid analysis using high-performance liquid chromatography

**DOI:** 10.1155/S1463924683000115

**Published:** 1983

**Authors:** D. C. Turnell, J. D. H. Cooper

**Affiliations:** Department of Biochemistry Coventry and Warwickshire Hospital Stoney Stanton Road Coventry CV1 4FH UK


Journal of Automatic Chemistry, Volume 5, Number 1 (January–March 1983), pages 36–39

### Automated pre-column derivatization and its application to amino-acid analysis using high-performance liquid chromatography

**D. C. Turnell and J. D. H. Cooper**

*Department of Biochemistry, Coventry and Warwickshire Hospital, Stoney Stanton Road, Coventry CV1 4FH, UK*

### Introduction

Although chemical modification of analytes is a well-accepted adjunct to chromatography, little attention had been paid to the automation of pre-column derivatization techniques. An automated pre-column system would offer advantages when analyses are performed using labile derivatives; and also when the time between injections is long and so staff time spent on achieving maximum analytical output is less than optimal.

Both of these features exist with the analysis of amino-acids in physiological samples using pre-column 0-phthalaldehyde/2-mercaptoethanol (OPA/MCE) derivatization and reversed-phase high-performance liquid chromatography (HPLC) [1]. These fluorescent derivatives are labile [1 and 2] and therefore have to be prepared in a precise manner immediately before injection onto the column.

A system is described for automatically sampling, derivatizing and injecting specimens onto an HPLC and analysing for amino-acids. Furthermore, this system may be operated overnight—capitalizing on the reduced chromatographic run time when compared with amino-acid analysis using ion-exchange post-column derivatization methods [3].

### Materials and methods

### *Instrumentation*

The sample derivatization system comprised a Magnus M7100 sampler fitted with a pneumatically-operated Rheodyne 7010 valve (Magnus Scientific Ltd, Aylesbury, UK), a Technicon AA1 pump fitted with a 0·32 ml/min and a 0·10 ml/min pump tube and associated autoanalyser components (Technicon Instruments Company Ltd, Basingstoke, UK). Chromatographic separations were performed using an Altex 420 gradient HPLC with a 150 × 4·6 mm i. d. column pre-packed with 5 $\mu$m diameter Ultrasphere ODS (Anachem Ltd, Luton, UK) and a Schoeffel FS970 fluorescence detector (Kratos, Manchester, UK). Chromatographic data was processed by a SP4100 computing integrator (Spectra-Physics Ltd, St. Albans, UK).

*Modifications to M7100 sampler*

The original minipump supplied with the sampler was disconnected. The 240 VAC power-supply to this pump was fed to a 240 VAC–110 VAC step-down transformer and, in turn, to the Technicon AA1 pump. The 110 VAC supply line was also fed to a two-pole change-over octal relay (115 VAC), which controlled the supply to the pneumatics of the Rheodyne valve. This enabled simultaneous switching of the injection loop and control of the Technicon AA1 pump.

*Double-lumen probe*

Both of the axial nipples of a PT 9 connector (Technicon Instruments Company Ltd) were removed. One was replaced with a 3 cm length of a stainless-steel needle (0·8 mm i.d. × 1·2 mm

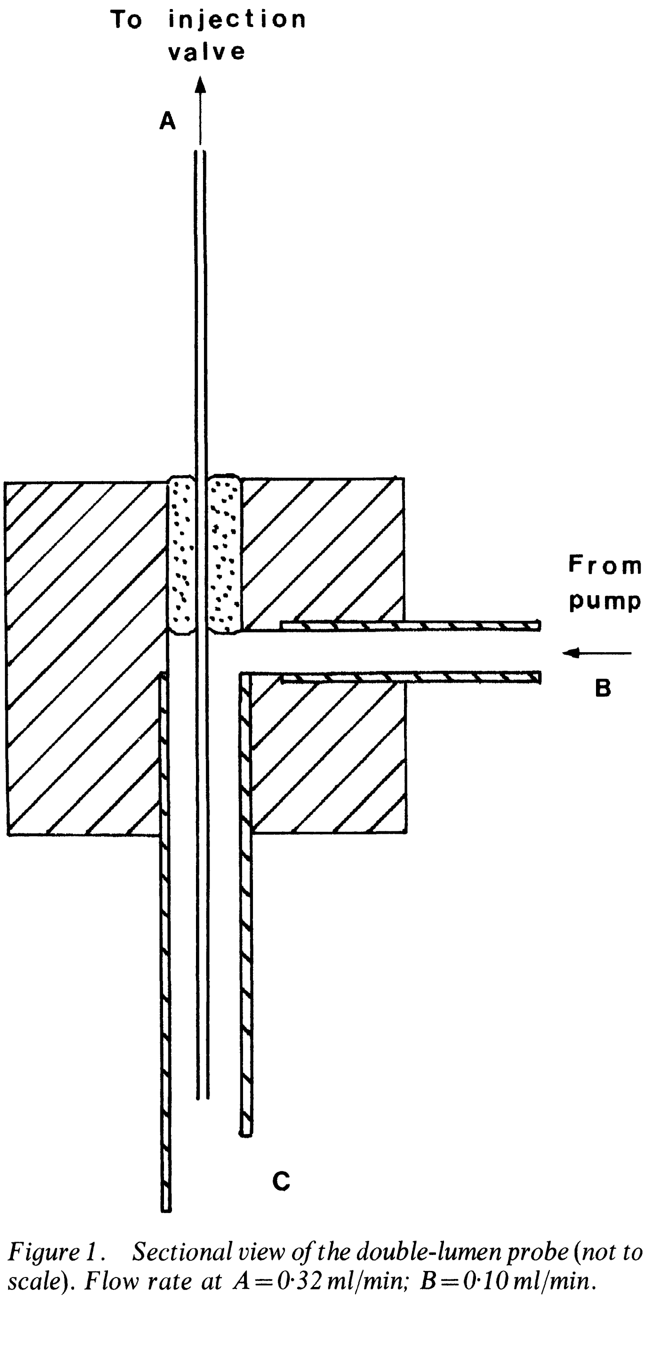

*Figure 1. Sectional view of the double-lumen probe (not to scale). Flow rate at A = 0·32 ml/min; B = 0·10 ml/min.*

o.d.). A stainless-steel capillary tube, 9 cm long (0·4 mm i.d. × 0·6 mm o.d.), replaced the other axial nipple so that one end was approximately 1 mm above the needle tip (see figure 1). The junction between the capillary tube and the top of the connector was filled with epoxy cement.

By using a larger flow rate at A (figure 1) than at B, the sample is drawn into the probe at C at a rate equal to the difference between the flow rate at A and B. The probe is connected to the injection valve with Teflon tubing (0·3 mm i.d. × 1·5 mm o.d.).

### *Reagents*

All amino-acids were obtained from the Sigma London Chemical Company Ltd, Poole, UK. Iodoacetic acid and 2-mercaptoethanol were purchased from the Aldrich Chemical Company, Gillingham, UK. Acetonitrile Far UV grade was obtained from Fisons Scientific Apparatus, Loughborough, UK. Unless otherwise stated, all other chemicals were analytical grade and obtained from BDH Chemicals Ltd, Poole, UK. Distilled deionized water was used for all reagent preparations.

*OPA/MCE reagent:* dissolve 500 mg of OPA (Sepramar grade) in 10 ml of methanol and dilute to 100 ml with a 400 mmol/l sodium borate buffer solution (pH 9·5). To this solution add 400 μl of 2-mercaptoethanol followed by a further 40 μl every three days. When stored in a brown-glass bottle this reagent was stable indefinitely at room temperature.

*Iodoacetic acid reagent:* dissolve 0·74 g of iodoacetic acid and 0·62 g of boric acid in approximately 50 ml of water. Adjust the pH to 9·5 with 2 mol/l sodium hydroxide solution and dilute to 100 ml with water. This reagent is stable indefinitely at room temperature.

*Acetonitrile precipitation reagent:* add 200 μl of 2-mercaptoethanol to 100 ml of acetonitrile. Prepare this reagent daily.

*Standard amino-acid solution:* the amino-acid standard was an aqueous 500 μmol/l solution of each amino-acid listed in figure 4.

*Internal standard solution:* the internal standard was an aqueous 1 mmol/l solution of homocysteic acid, homoserine and norvaline. Aliquots of both the standard and internal standard solutions were stored at −20°C.

### *Procedures*

### *Sample preparation*

20 μl of internal standard solution and 200 μl of the acetonitrite precipitation reagent was added to 20 μl of standard amino-acid solution, or specimen for analysis, in a polypropylene tube. The contents of all tubes were mixed in a vortex and centrifuged at 12 000 g for 2 min. 500 μl of the iodoacetic acid reagent was added to 100 μl of the supernatant solution in a polystyrene M7100 sampler cup.

### *Derivatization system*

Figure 2 shows a schematic diagram of the derivatization system. One cycle is shown in the timing diagram (figure 3)—it operates as follows: the M7100 sampler lowers the double-lumen probe into a sample cup, the AA1 Technicon pump is switched on and the injection valve turned to load. The sample is drawn into the probe where it mixes with the OPA/MCE reagent and passes to the injection valve. After 60 s the injection valve is rotated to inject and the AA1 Technicon pump switched off. This corresponds to the tubing between the probe and the outlet of the injection loop being purged six times. The sampler advances the sample plate to the next sample position. The

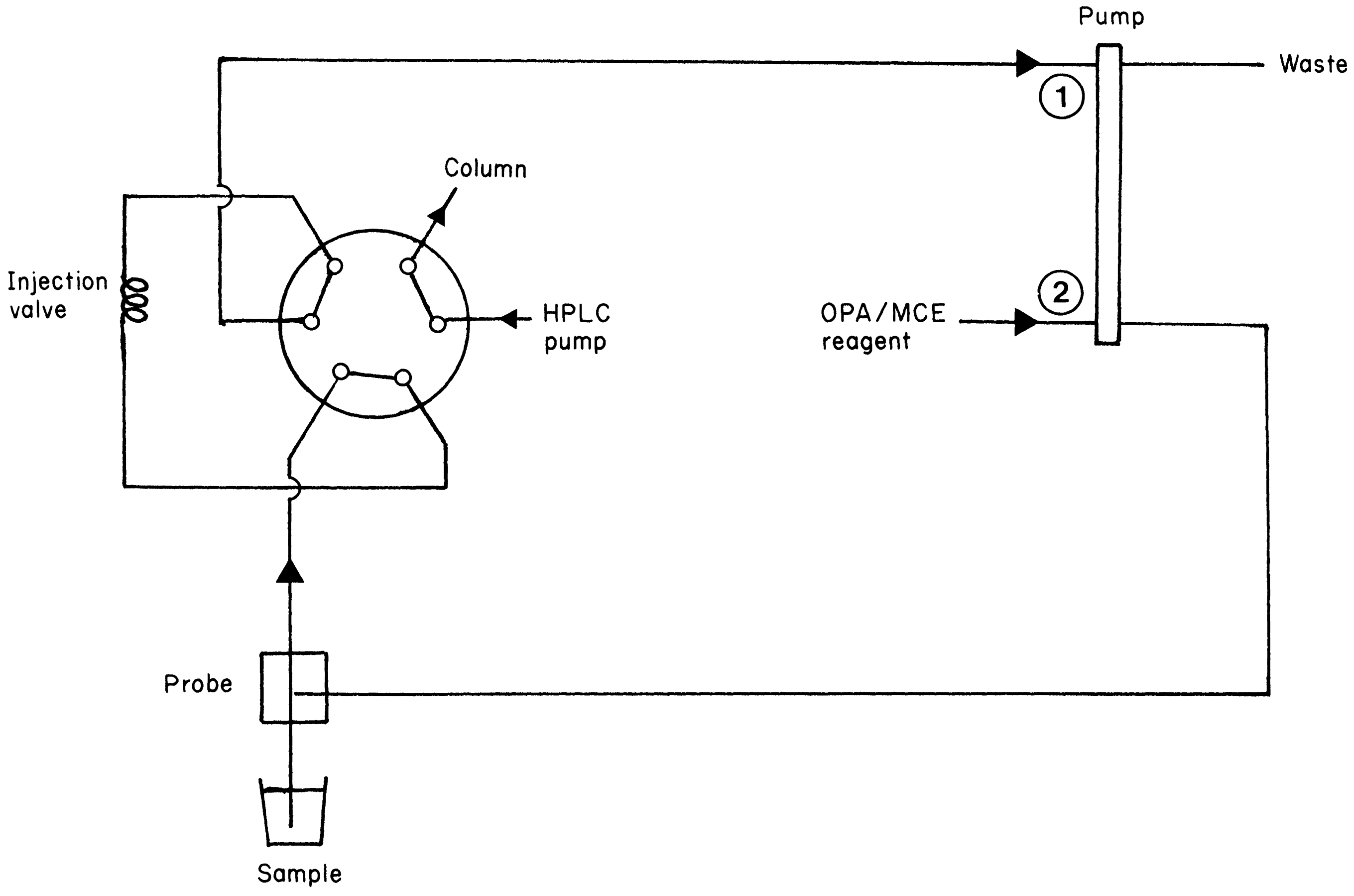

*Figure 2. Schematic diagram of the sample derivatization system. Flow rate of pump tube 1 = 0·32 ml/min; pump tube 2 = 0·10 ml/min.*

system remains in this state until the next sample is required.

*Chromatographic conditions and quantitation*

The chromatographic conditions used have been previously described [1]. Amino-acid derivatives were identified by their relative retention times and quantified by comparing their peak areas with that of homocysteic acid and the internal standard.

### System validation

Before the performance of the system derivatizing the amino-acids could be investigated, it was necessary to establish the stability of the samples treated with iodoacetic acid. The reproducibility of the system to sample, derivatize analytes and then quantify them was examined by determining the assay precision.

The degree of interaction between sequential samples was established by determining the sample carry-over of the system.

Because the amino-acid OPA/MCE derivatives are labile, any variance in the operation of the system would disproportionately increase the assay variance.

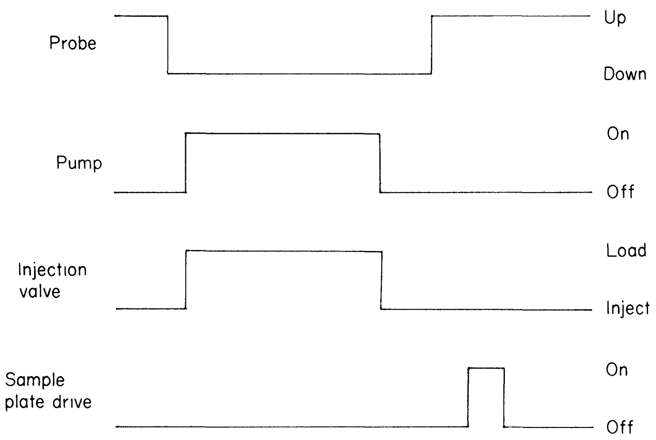

*Figure 3. Timing diagram of the sample derivatization system.*

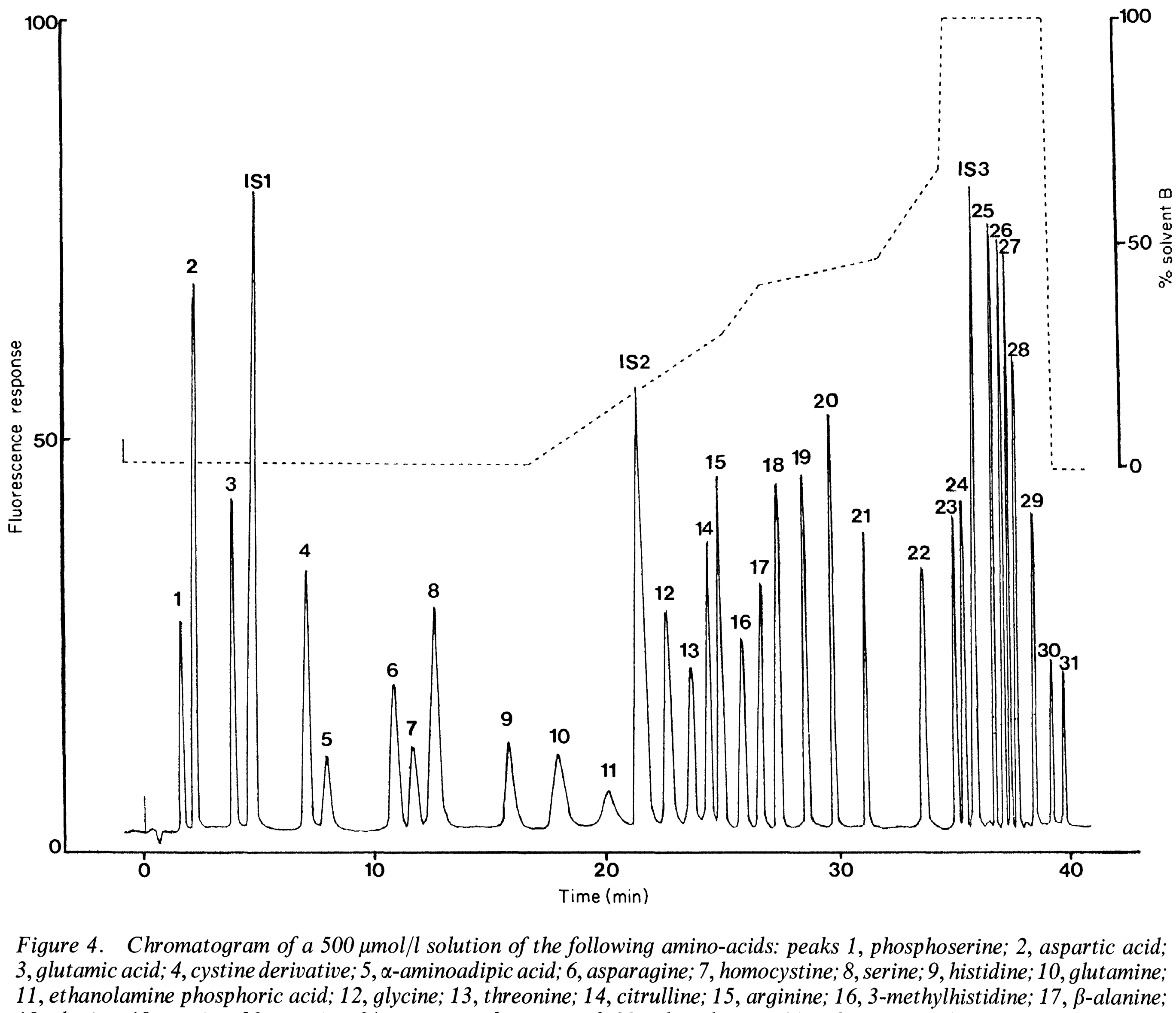

*Figure 4. Chromatogram of a 500 µmol/l solution of the following amino-acids: peaks 1, phosphoserine; 2, aspartic acid; 3, glutamic acid; 4, cystine derivative; 5, α-aminoadipic acid; 6, asparagine; 7, homocystine; 8, serine; 9, histidine; 10, glutamine; 11, ethanolamine phosphoric acid; 12, glycine; 13, threonine; 14, citrulline; 15, arginine; 16, 3-methylhistidine; 17, β-alanine; 18, alanine; 19, taurine; 20, tyrosine; 21, α-amino-η-butyric acid; 22, ethanolamine; 23, valine; 24, methionine; 25, tryptophan; 26, phenylalanine; 27, isoleucine; 28, leucine; 29, hydroxylysine; 30, ornithine; 31, lysine. IS1 = homocysteic acid, IS2 = homoserine, IS3 = norvaline. The broken lines indicate solvent gradient composition.*

*Stability of samples treated with iodoacetic acid*

A pooled human serum specimen was prepared for analysis using the sample treatment procedure described. A 40 μl aliquot of this sample was added to 20 μl of OPA/MCE reagent in a polypropylene tube. The contents of the tube were mixed, and 20 μl immediately injected onto the column using a 7125 Rheodyne 20 μl loop injection valve (from Anachem Ltd). This manual derivatization and injection procedure was repeated on the same sample every 2 h for 24 h. The stability of a standard and urine specimen were investigated in the same manner. Over the period, no significant variation was observed in the concentrations of the amino-acids.

*Derivatization precision*

The performance of the derivatization system was examined by estimating the within-run assay variance, with and without reference to the internal standard (see table 1). The amino-acids quantified were selected to provide retention times ranging throughout the chromatogram.

These precisions represent the reproducibility of (*a*) the volume of sample aspirated; (*b*) the mixing of the sample with OPA/MCE reagent; (*c*) the yield of the derivatives; (*d*) the time delay between sampling and loading; and (*e*) the filling of the injection valve loop.

*Sample carry-over*

A 1·0 mmol/l standard amino-acid solution was prepared for sampling. Using a cycle time of 5 min (to ensure that the system was completely filled with derivatized sample), the standard solution was assayed without the presence of internal standard. Immediately afterwards, a water sample was aspirated with a cycle time of 15 s and the amino-acids quantified again. This procedure was repeated increasing the cycle times of the water sample (see table 2). The cycle time for the system was set at 60 s when negligible carry-over occurred.

*Table 1. Precision of the derivatization system.*

| | | Coefficient of variation of assay ($N = 15$) | |
|---|---|---|---|
| Retention time (min) | Amino-acid | Without reference to internal standard | With reference to internal standard |
| 2·3 | 2, Aspartic acid | 1·6 | 2·2 |
| 4·8 | ISI homocysteic acid | 1·7 | |
| 12·5 | 8, Serine | 1·2 | 0·9 |
| 23·5 | 13, Threonine | 1·2 | 1·4 |
| 30·0 | 20, Tyrosine | 1·2 | 1·7 |
| 35·3 | 24, Methionine | 1·2 | 1·3 |

*Table 2. Sample carry-over from a 1·0 mmol/l standard after aspirating water for various cycle times.*

| Cycle time (s) | Carry-over (%) |
|---|---|
| 15 | 67 |
| 25 | 8 |
| 35 | 1·9 |
| 50 | 0·3 |
| 60 | 0·3 |

### Discussion

Ideally, derivatization of analytes before or after chromatographic separation should be avoided because it imposes further potential errors in techniques. If, however, for such reasons as detection, specificity or chromatographic resolution, it becomes necessary to derivatize analytes, the method used should be one with minimal adverse effect on the performance of the analytical technique. Pre-column derivatization is superior to post-column derivatization in that its effect on analytical performance is minimal for the following reasons: (*a*) it does not involve an increase in path-length between the column and detector and therefore does not decrease separation efficiency; and (*b*) post-column derivatization equipment has to be engineered to operate continuously within narrow tolerance limits because internal standards cannot be incorporated to correct for all possible variations in performance.

The design of the pre-column derivatization system had to fulfil two basic requirements; firstly, the ratio of the amount of sample loaded to the amount of sample consumed had to be large; and, secondly, the continuous-flow system had to operate discontinuously with a short cycle time in order to conserve reagents.

The double-lumen probe permits samples to be aspirated, well mixed with reagents and transferred directly to the injection valve. This minimizes the volume of tubing that the sample passes through and thus reduces the volume of sample required to purge the system of the previous specimen.

In conventional continuous-flow systems, flow rates take some time to stabilize due to the compressibility of the air segmentation used to assist mixing and minimize sample diffusion. If air segmentation was to be used in this application, the air would have to be removed before the injection and would grossly extend the sampling time. The unsegmented stream used in this system permits rapid stabilization of flow rates, with a low volume and simple peristaltic tubing configuration.

Because the double-lumen probe is connected to both the inlet and the outlet of the peristaltic pump, the inherent pulsing observed with this instrument is amplified at the probe inlet (see C in figure 1). To reduce this effect, it is necessary that the flow rate ratio A/B (figure 1) be no less than 3. Also, to obtain acceptable precision the injection valve has to be turned from load to inject while the sample stream is flowing through the loop (table 1). This was achieved in this system with the sampler modification.

The precision of the system in the absence of the internal standard (table 1) demonstrates that stable flow rates exist and that, despite the absence of air segmentation, the mixing of the sample and reagent is adequately reproducible and carry-over is minimal. Moreover, the larger coefficients of variation obtained when the internal standard was used for the quantitation indicates that the errors of chormatography and integration were greater than the errors of sampling and derivatization. The double-lumen probe further reduces the time taken for the sample to pass to the injection loop by introducing it immediately into the sample/reagent stream. Even when derivatization is not required, this feature of the double-lumen probe is useful for rapidly loading samples automatically onto a liquid chromatograph.

When the double-lumen probe is used in conjunction with an unsegmented-flow technique, a minimum amount of sample may be derivatized and loaded in the shortest possible cycle time in a discontinuous mode.

### References

1. Turnell, D. C. and Cooper, J. D. H., *Clinical Chemistry*, **28** (1982), 527.
2. Lindroth, P. and Mopper, K., *Analytical Chemistry*, **51** (1979), 1667.
3. Moore, S., Spackman, D. H. and Stein, W. H., *Analytical Chemistry*, **30** (1958), 1185.

